# The inverted U-shaped relationship between knowledge diversity of researchers and societal impact

**DOI:** 10.1038/s41598-022-21821-0

**Published:** 2022-11-03

**Authors:** Gaofeng Wang, Yetong Gan, Haodong Yang

**Affiliations:** School of Humanities and Social Sciences, University of Science and Technology of China, Hefei, China

**Keywords:** Psychology, Human behaviour

## Abstract

With the increasing importance of interdisciplinary research, some studies have focused on the role of reference diversity by analysing reference lists of published papers. However, the relationship between the knowledge diversity of collaborating team members and research performance has been overlooked. In this study, we measured knowledge diversity through the disciplinary attributes of collaborating authors and research performance (understood as societal impact) through altmetric data. The major findings are: (1) The relationship between interdisciplinary collaboration diversity and societal impact is not a simple linear one, showing an inverted U-shaped pattern; and (2) As the number of collaborative disciplines increases, the marginal effects diminish or even become outweighed by the costs, showing a predominance of negative influences. Hence, diversity in interdisciplinary collaboration does not always have a positive impact. Research collaborations need to take into account the cost issues associated with the diversity of member disciplines.

## Introduction

Given that scientific collaboration is currently the main mode of knowledge production, its value is enormous. Co-authored papers are more frequently cited and have greater societal impact than ever before^1,2^. Research collaborations not only drive innovation and disciplinary breakthroughs but also contribute significantly to solving complex and important social problems, thus becoming one of the most important forms of knowledge creation and management. Against this background, the driving forces behind collaborations are receiving increasing attention from researchers. Studies have found a significant linear relationship between collaboration diversity, which primarily refers to the degree of variation among team members’ characteristics, and publication in high impact factor journals; collaboration diversity positively predicts citation rates^3–5^. Specifically, collaboration diversity’s ability to increase the visibility of research (through more diverse social networks) may increase academic impact^6^. If these factors are excluded, it still suggests that research papers with higher collaboration diversity are more popular with peer reviewers^7^. In today’s rapidly evolving knowledge economy, researchers aim to maximise the benefits of extensive scientific collaborations across different organisations or countries^8^. However, in the context of increasing team sizes, it remains unclear whether continually increasing diversity always plays a positive role. While diversity promotes complementary skills and knowledge reconfiguration among members, it also increases cognitive costs and affective biases to some extent^9^, which may reduce the efficiency of collaboration. In the relevant literature, the marginal benefits of collaboration diversity for research performance are yet to be explored in depth. Such an investigation may provide an empirical basis and insights for understanding scientific activity patterns and research team management, and this is the question that the present study seeks to address.

Collaboration diversity primarily refers to the degree of variation among members’ characteristics. Previous studies have examined the relationship between collaboration diversity and research performance in terms of research members’ characteristics, such as institution, ethnicity, and country^10–13^. Nevertheless, if we examine collaboration as a research activity based on knowledge interaction, the diversity of research participants in terms of their knowledge backgrounds is particularly crucial. As a typical example of knowledge diversity in research collaboration, interdisciplinary collaboration involves researchers from different disciplines using a problem-oriented approach to conduct academic research through multiple forms of collaboration. This approach allows them to integrate multidisciplinary forms of knowledge and produce innovative research results^14^. From the perspective of scientific communication, it is the scientific community that brings its specialised knowledge, research logic, and way of thinking into the process of scientific activity and thereby accomplishes a breakthrough in interdisciplinary research.

Prior studies examining the relationship between interdisciplinary collaboration and research performance have mainly used academic impact as an indicator of research performance. This may represent a limitation, given that they excludes civil society and public discourse, which were identified as an essential part of current high-level knowledge production (“Mode 3”)^15^. The participation of the public activates new ideas for solving problems and likewise accelerated the practice of scientific research. Along these lines, Carayannis and Campbell^16^ added “civil society” to the “academia-industry-government” model to achieve an ecological balance of innovation in the public interest. They constructed a quadruple helix model of the dynamics of knowledge production, which called for a shift in the focus of knowledge evaluation systems from academic to societal impact. For example, the Research Excellence Framework (REF) in the UK or Moed and Halevi^17^ advocated a multidimensional evaluation system that separated the impact of science into academic, societal, economic and other. Among them, societal impact broke the limitations of recognition by the academic community only. It emphasized the degree of interest which non-academic workers, such as policy makers and people in society, take in research^18^. As an important channel of communication and dissemination between academia and society at large, social media facilitated the behavior of interaction between them. Based on that societal impact was generated^19^. In this context, the quantitative analysis of altmetrics based on social network data has also emerged. It was considered “the creation and study of new social network-based metrics to analyse academic intelligence”^20^, reflecting the level of public interest in scientific research. To some extent, this provided a measure for societal impact-oriented research evaluation systems^21,22^. Altmetrics were more useful for uncovering general patterns than qualitative case study research which were widely applied in research on the evaluation of the societal impact of research outcomes^23,24^.

Only a few studies have explored the relationship between knowledge or disciplinary diversity and societal impact in recent years, and the results have been controversial. Some scholars have found that the usefulness of papers increases in proportion with their disciplinary diversity^25–27^. Conversely, Shi et al.^28^ divided the highly cited papers in the field of library intelligence into high, medium, and low interdisciplinary groups and found that interdisciplinarity was highly negatively correlated with societal impact. It is important to note that the above-mentioned studies measured the disciplinary diversity of papers through references. They focused on the disciplinary origin and related structure of the research participants, which helped characterise the paths of the knowledge flow^29,30^. Such studies highlighted interdisciplinary citation patterns; they did not directly reflect the patterns of communication and collaboration between research participants from different disciplinary backgrounds^31^. Critically, the relationship between disciplinary diversity and societal impact in interdisciplinary collaboration remains to be explored.

In summary, this study proposes to conduct research on the relationship between diversity in interdisciplinary collaboration and societal impact. To address this question, we measured the level of interdisciplinary collaboration diversity using the disciplinary attributes of authors of publications (details in “Methods”) and reflected the societal impact of the co-authored papers through altmetrics.

### The relationship between interdisciplinary collaboration diversity and societal impact

Understanding the essence of the relationship between knowledge diversity in interdisciplinary collaboration and societal impact involves investigating the impact of collaboration diversity on research performance^32^. Most studies suggest that collaboration diversity has a positive effect on research output, which encourages research institutions to actively engage in diverse collaborations and researchers to seek new partners^5,11,33,34^. However, other studies have found that heterogeneity in team members” knowledge hinders creativity and reduces performance^9,35^. These inconsistent findings may reflect the fact that this relationship is non-linear.

Theoretically, knowledge diversity among collaborating members is often considered a double-edged sword. On the one hand, in terms of information processing, knowledge diversity challenges existing knowledge structures, stimulates creativity and divergent thinking, and facilitates knowledge reconstruction by identifying and integrating knowledge from different fields^9,36^. Just as the weak ties hypothesis emphasises the importance of different resources, richer and more diverse information contributes to improved collaborative performance^37,38^. On the other hand, increasingly heterogeneous knowledge can exacerbate the knowledge boundaries among team members and increase the cognitive costs required for shared knowledge construction^39^.

Similar trends were observed at the level of team interactions. Highly heterogeneous teams experienced some degree of conflict when collaborating because of their different perceptions of the task^40,41^. Moderate levels of task conflict are more conducive to complex issue resolution than excessively high or low levels^42^. Simultaneously, researchers’ pride in their own discipline can prompt protective behaviour in the domain of knowledge. Moreover, the use of technical discipline-specific terms may deepen communication barriers and cause other challenges^43^. The effectiveness of team communication and decision-making decreases as diversity increases^44^.

It is clear from the above that as knowledge diversity increases, the advantages do not always outweigh the disadvantages; in terms of diversity, there can be “too much of a good thing”. There is a threshold of positive effects beyond which undesired outcomes are produced, resulting in an inverted U-shaped non-linear relationship^45,46^. Accordingly, we defined Hypothesis as follows:

The impact of knowledge diversity in the relationship between interdisciplinary collaboration and societal impact forms an inverted U-shape. As diversity increases, societal impact reaches a peak and then tends to decline.

## Results

### Descriptive statistics and correlation analysis

The results of the descriptive statistics and correlation analysis of the variables revealed significant positive correlations between Tweets mentioned counts and the number of subjects (Table 1). To avoid the influence of outliers on the results, the data were Winsorized to trime the dependent variables at the 99% quantile. The final sample quantity was 1539. Related results showed that the correlation coefficients were below the critical value of 0.7, indicating there were no serious collinearity issues between the variables. Accordingly, we proceeded with further hypothesis testing and regression analysis.

**Table 1:** Descriptive statistics and correlation coefficients.

Variables	1	2	3	4	5	6	7	8	9	10	11	12
1. Altmetric attention score	1.000											
2. Tweets mentioned count	0.735	1.000										
	(0.000)											
3. Subject count	0.157	0.209	1.000									
	(0.000)	(0.000)										
4. Organization count	0.206	0.187	0.662	1.000								
	(0.000)	(0.000)	(0.000)									
5. Author count	0.157	0.149	0.428	0.761	1.000							
	(0.000)	(0.000)	(0.000)	(0.000)								
6. Journal	0.006	− 0.053	0.008	− 0.038	− 0.033	1.000						
	(0.807)	(0.036)	(0.752)	(0.139)	(0.188)							
7. Time lag	0.056	0.035	− 0.027	− 0.018	− 0.034	0.007	1.000					
	(0.027)	(0.169)	(0.279)	(0.472)	(0.180)	(0.783)						
8. Fields_Physical sciences and engineering	− 0.135	− 0.300	− 0.262	− 0.096	− 0.043	0.064	− 0.025	1.000				
	(0.000)	(0.000)	(0.000)	(0.000)	(0.091)	(0.012)	(0.332)					
9. Fields_Life and earth sciences	0.265	0.255	0.093	0.032	− 0.035	0.015	0.009	− 0.360	1.000			
	(0.000)	(0.000)	(0.000)	(0.202)	(0.170)	(0.553)	(0.726)	(0.000)				
10. Fields_Biomedical and health sciences	− 0.095	0.127	0.225	0.066	0.093	− 0.056	0.045	− 0.635	− 0.195	1.000		
	(0.000)	(0.000)	(0.000)	(0.009)	(0.000)	(0.028)	(0.078)	(0.000)	(0.000)			
11. Fields_Social sciences and humanities	0.213	0.149	0.009	0.004	− 0.043	0.039	0.017	− 0.132	0.077	− 0.112	1.000	
	(0.000)	(0.000)	(0.718)	(0.877)	(0.094)	(0.120)	(0.514)	(0.000)	(0.003)	(0.000)		
12.Fields_Mathematics and computer science	0.044	0.005	− 0.067	− 0.064	− 0.043	− 0.037	0.019	0.060	− 0.066	− 0.079	0.020	1.000
	(0.085)	(0.840)	(0.009)	(0.012)	(0.092)	(0.147)	(0.449)	(0.018)	(0.010)	(0.002)	(0.422)	
Mean	281.75	171.03	4.421	7.979	14.472	0.452	1492.96	0.342	0.273	0.506	0.032	0.023
Standard deviation	360.22	226.82	2.768	9.368	18.932	0.498	103.057	0.475	0.446	0.5	0.177	0.15

P-value are in parentheses. Journal and all fields are both dummy variables.

### Regression analysis

Given that the variance of the dependent variable was larger than the mean and showed an over-dispersed distribution (i.e. it did not meet the requirements of a general multiple linear regression), we modified the model using a negative binomial regression applicable to asymmetric datasets. The variance inflation factor (VIF) for each variable was less than 10, excluding the possibility of multicollinearity between the independent and control variables. The regression Eq. (1) is as follows:

1 $$TMC={\beta }_{0}+{\beta }_{1}\left(\mathrm{Subject count}\right)+{\beta }_{2}\left({\mathrm{Subject count}}^{2}\right)+{\beta }_{3}\left(\mathrm{Organization count}\right)+{\beta }_{4}\left(\mathrm{Author count}\right)+{\beta }_{5}\left(\mathrm{Journal}\right)+{\beta }_{6}\left(\mathrm{Time lag}\right)+{\beta }_{7}\left({\mathrm{Fields}}_{\mathrm{PSE}}\right)+{\beta }_{8}\left({\mathrm{Fields}}_{\mathrm{LES}}\right)+{\beta }_{9}\left({\mathrm{Fields}}_{\mathrm{BHS}}\right)+{\beta }_{10}\left({\mathrm{Fields}}_{\mathrm{SSH}}\right)+{\beta }_{11}\left({\mathrm{Fields}}_{\mathrm{MCS}}\right)$$

Model 1 showed the regression results for all the control variables on societal impact. Models 2 and 3 tested the regression results for the linear term and the quadratic term for the societal impact on diversity in interdisciplinary collaboration, respectively (Table 2). The 95% confidence interval for the alpha value of the negative binomial regression model did not include zero, indicating that the model rejected the original hypothesis of “alpha = 0” at the 5% significance level; hence, the negative binomial regression model was basically acceptable^47^.

**Table 2:** Regression analysis results.

Variables	Model 1		Model 2		Model 3	
	*M*	*SE*	*M*	*SE*	*M*	*SE*
**Control variables**						
Institution count	0.013**	0.005	0.006	0.007	0.012*	0.007
Author count	0.005*	0.003	0.006**	0.003	0.004*	0.002
Journal	− 0.207***	0.072	− 0.214***	0.071	− 0.216***	0.071
Time lag	0.0003	0.0003	0.0003	0.0003	0.0003	0.0003
Fields_*Physical sciences and engineering*	− 0.584***	0.126	− 0.578***	0.124	− 0.583***	0.124
Fields_*Life and earth sciences*	0.678***	0.091	0.655***	0.090	0.634**	0.089
Fields_*Biomedical and health sciences*	0.388***	0.111	0.353***	0.109	0.346***	0.108
Fields_*Social sciences and humanities*	0.836***	0.146	0.838***	0.146	0.822***	0.146
Fields_*Mathematics and computer science*	0.862***	0.323	0.843***	0.323	0.833***	0.322
**Independent variables**						
Subject count			0.029	0.019	0.099***	0.031
Subject count^2^					− 0.006***	0.002
Constant	4.187***	0.483	4.131***	0.481	3.958***	0.477
Wald chi^2^	267.85***		283.70***		295.44***	
AIC/BIC	18,420.86/18,479.58		18,418.83/18,482.89		18,410.12/18,479.52	
Pseudo R^2^	0.024		0.024		0.025	
Alpha 95%CI	[0.941, 1.086]		[0.939, 1.084]		[0.934, 1.078]	

***p < 0.01, ** *p* < 0.05, * *p* < 0.1; Number of observations = 1539.

**Figure 1:**
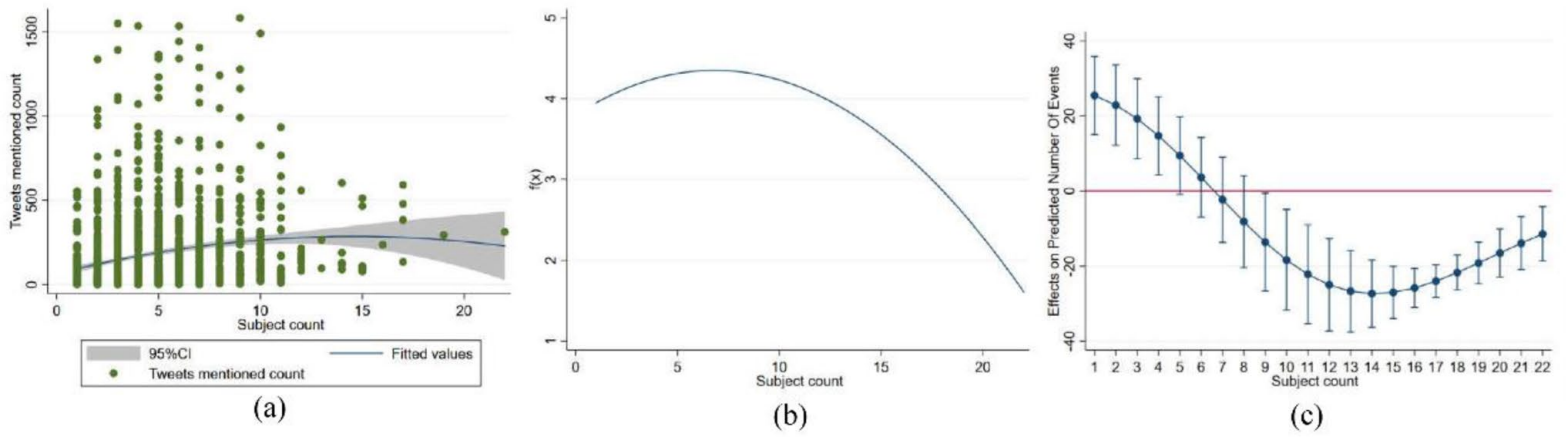
**(a)** Scatter plot and fitted curve of number of Tweets mentioned count vs. subject count **(b)** Regression function graph **(c)**Average marginal effects of subject count with 95% CIs.

As seen in Fig. 1a, the fitted curve for Tweets mentioned count to subject count showed an inverted U-shaped trend. This study adopted the AIC and BIC statistic to determine the fit of the quadratic model versus the linear model. When comparing the two, a smaller AIC or BIC means a better model fit. It was found that the quadratic model revealed more patterns in the data than the linear model (See Models 2 and 3 of Table2 for detailed results). Likewise, the Pseudo R^2^ data for the three models supported that the model fit was better for the quadratic terms.

The results showed that the linear term for knowledge diversity in interdisciplinary collaboration positively predicted the number of Tweets, while the regression results for the quadratic term were negative and significant (*β*_1_ = 0.099, *p* < 0.001, *β*_2_ = − 0.006, *p* < 0.001), thus supporting Hypothesis. To further test the inverted U-shaped hypothesis, we adopted the utest command in Stata and found that the extreme value points were within the data limits (1 < 6.62 > 22), and the 95% confidence interval was [4.745, 8.892]. As such, the null hypothesis of no U-shape could be rejected at the 5% significance level (t = 3.85, *p* < 0.001). The trends in collaboration diversity and societal impact are plotted against the negative binomial regression results in Fig. 1b.

Figure 1c showed a further analysis of the marginal effects. It was found that as the diversity of interdisciplinary membership increases, the marginal effect on societal impact continues to diminish and even becomes negative. At an average number of disciplines of 6.6, the equilibrium point between costs and benefits is reached. However, the costs then begin to outweigh the benefits and the slope becomes negative.

### Robustness tests

**Figure 2:**
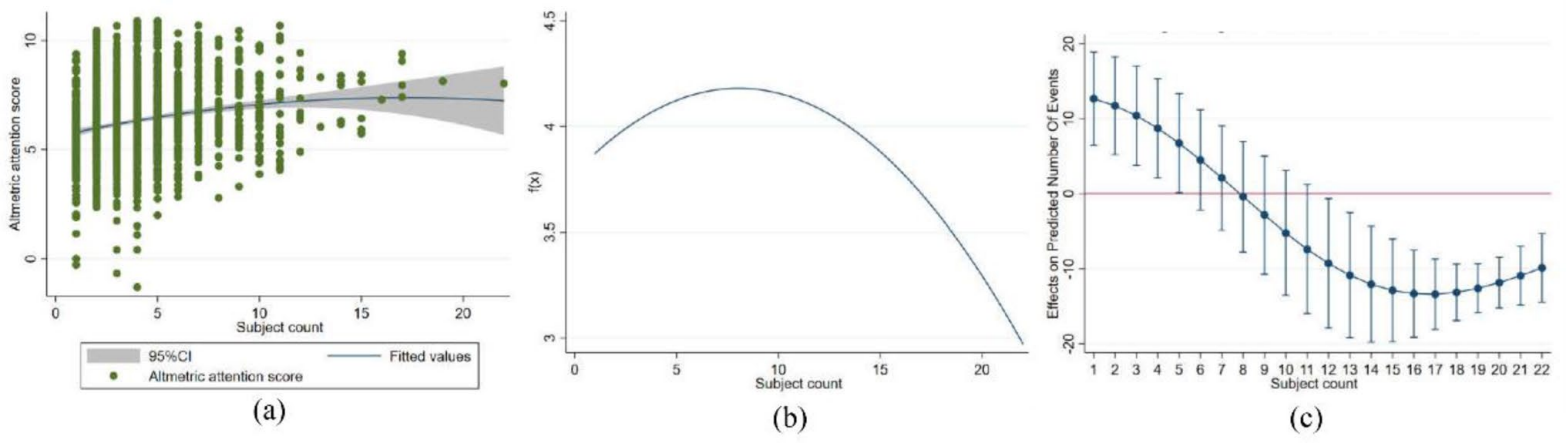
**(a)** Scatter plot and fitted curve of number of altmetric attention score vs. subject count **(b)** Regression function graph **(c)**Average marginal effects of subject count with 95% CIs.

To verify the regression results, we conducted robustness tests using another important measure of societal impact: altmetric attention score (Table 3 and Fig. 2a,b). Since altmetric attention score was a non-integer data, it was not rigorous to employ negative binomial regression analysis. This study adopted boxcox transformed dependent variable data for OLS regression analysis. The results showed that the linear term for collaboration diversity positively predicted altmetric attention score, while the regression results for the quadratic term were negative and significant (*β*_1_ = 0.162, *p* < 0.01, *β*_2_ = − 0.012, *p* < 0.01).

**Table 3:** Regression analysis results.

Variables	Model 1		Model 2		Model 3	
	*β*	*SE*	*β*	*SE*	*β*	*SE*
**Control variables**						
Institution count	0.038***	0.009	0.031***	0.011	0.042***	0.012
Author count	0.006	0.004	0.006*	0.004	0.005	0.004
Journal	− 0.127	0.082	− 0.134	0.082	− 0.127	0.082
Time lag	0.001***	0.0004	0.001***	0.0004	0.001***	0.0004
Fields_*Physical sciences and engineering*	− 0.363**	0.154	− 0.351**	0.154	− 0.356**	0.153
Fields_*Life and earth sciences*	0.982***	0.117	0.970***	0.117	0.949***	0.116
Fields_*Biomedical and health sciences*	− 0.217	0.135	− 0.244*	0.135	− 0.245*	0.134
Fields_*Social sciences and humanities*	1.814***	0.195	1.814***	0.197	1.790***	0.198
Fields_*Mathematics and computer science*	0.885***	0.334	0.887***	0.332	0.899***	0.333
**Independent variables**						
Subject count			0.029	0.021	0.162***	0.042
Subject count^2^					− 0.012***	0.003
Constant	4.266***	0.592	4.160***	0.593	3.798***	0.591
AIC/BIC	5782.29/5835.68		5782.253/5840.98		5769.82/5833.89	
R^2^	0.202		0.203		0.211	
Root MSE	1.578		1.578		1.571	

Robust standard errors are in parentheses; ***p < 0.01, **p < 0.05, *p < 0.1.

To further test the inverted U-shaped hypothesis, we adopted the utest command in Stata and found that the extreme value points were within the data limits (1 < 6.823 > 22), and the 95% confidence interval was [5.045, 9.865]. As such, the null hypothesis of no U-shape could be rejected at the 5% significance level (t = 3.18, *p* < 0.001). Furthermore, the results of the marginal effects analysis similarly indicated that the instantaneous slope of subject diversity and societal impact shifted from positive to negative. This suggested that when the number of disciplines exceeded the optimal size, the marginal costs outweigh the marginal effects and the negative impact becomes dominant (See Fig. 2c).

## Discussion and conclusion

### Main findings

Grounded in the question of whether diversity in collaboration is always optimal, this study explored the relationship between collaboration diversity and societal impact in interdisciplinary research, and the role of cognitive distance. Based on a literature review and theoretical analysis, we suggested that this relationship may take the form of an inverted U-shaped pattern. We used a sample of co-authored studies published in *Nature* and *Science* to test the hypotheses. The results indicated a significant inverted U-shaped relationship between the number of cross-disciplinary collaborators and both the number of times the paper was mentioned on Twitter and the altmetric attention score. This implied that as the knowledge diversity of the collaboration members increases, societal impact tends to decline after it reaches a certain peak.

Current mainstream research has dissected the benefits of collaboration diversity from a variety of perspectives. Philosophical explanations can be traced back to Aristotle’s famous defence of the “wisdom of the multitude”: “Hence the many are better judges than a single man of music and poetry; for some understand one part, and some another, and among them they understand the whole”^48^. This emphasises the role of diversity in facilitating collective decision-making. Furthermore, cognitive neuroscience experiments have shown that the proximity of other people’s ideas effectively enhances interpersonal brain synchronisation, thereby increasing team creativity and vice versa^49^. Contrastingly, some meta-analytic studies have found small or zero effect sizes for the positive relationship between demographic or cultural diversity and team performance^50,51^. Hence, it is evident that logical deduction and empirical studies continue to note both the advantages and disadvantages of collaboration diversity. This controversy may indicate that the advantages of collaboration diversity have their own specific scope and boundary conditions^52^. This study confirms, from an intellectual context, that the benefits of cooperative diversity may be reduced after a certain peak due to excessive costs. In scientific research, where divergence and convergence processes alternate, diversity is essential if scientists are to produce novel and rigorous results^53^; it is also important to pay attention to the cost of too much diversity and avoid inefficiency and the phenomenon of “too many cooks spoiling the broth”.

It is interesting to note that this finding is similar to the optimal scale effect found in recent years in scientific collaboration. Price’s famous prediction^54^ that scholarly publications will “move steadily toward an infinity of authors per paper” is being borne out, with the “scientist-as-lonely genius” myth becoming further detached from reality^55,56^. In fact, the benefits of team size were shown not to follow a linear growth pattern. With the continued “inflation” of collaboration size, citation rates appeared the tendency of reduction, which presented an inverted U-shaped relationship^7,57,58^. As Wu et al.^13^ found, large teams tend to develop existing science and technology more than small teams, resulting in fewer disruptive innovation breakthroughs. Increased team size is often accompanied by greater diversity among team members^58^, introducing more collective intelligence and innovative perspectives. There are exceptions, such as very diverse small groups and large, highly homogenous teams. The key question is whether size or diversity is more important in the relationship between interdisciplinary collaborative research and research performance. Collective intelligence research has focused on this relationship, with Condorcet’s jury theorem predicting that heterogeneous team composition may be more important when faced with highly complex decisions which are susceptible to bias^59^. To improve the accuracy of collective decision-making, a high level of diversity needs to be maintained if team size is to be continuously increased^60^. Despite controlling for the variable of team size, the present study still found an inverted U-shaped distribution in the relationship between collaboration diversity and societal impact, indicating the relatively greater importance of diversity in collective decision-making performance compared to team size. Zhu et al.^58^ discovered that research team members’ own research diversity played a moderating role in the relationship between team size and the influence of the research. In any case, our conclusions need to be interpreted with caution, as the relationship between size and diversity may be reversed under other conditions. For example, in real-world social activities, the performance of collective decision-making depends on a variety of factors, such as average individual accuracy and decision bias^59,61^.

### Implications

In theoretical terms, this study focused on the performance of collaboration diversity from the perspective of knowledge diversity in interdisciplinary collaboration. As an important cognitive basis for scientific collaboration, knowledge diversity among team members is a key element that influences innovation performance. In contrast to previous studies that have emphasised the value of collaboration diversity, this study quantitatively verified that collaboration diversity is a double-edged sword, peaking in the number range of 6 to 7, after which its negative effects may outweigh the positive ones.

In current society, the promotion of scientific and technological innovation through interdisciplinary collaboration has become a major concern for national governments and research institutions. This study provides important insights into the formation of research teams and knowledge management. It is important to be aware of the differences in the disciplinary backgrounds among team members and to try to optimise the level of disciplinary diversity. During collaboration, research teams, especially those working at the intersection of the humanities and sciences, need to be aware of potential communication barriers and conflicts among members.

### Limitations and future research

Note that while research on societal impact has widely used altmetric as indicators, we are aware of the limitations of measuring societal impact through analysis of social media activity. The findings of this study need to be validated with multiple sources of data and research methods. For example, societal impact is not only measured by the level of attention but also by the content. It is necessary to explore metrics for quantifying the valance of social evaluation. Furthermore, in addition to diversity and differences in disciplinary distances, interdisciplinary collaboration also involves disparity and balance. It is necessary for future research to reveal the patterns in interdisciplinary collaboration from more dimensions.

As a major part of scientific activity, scientific collaboration is a dynamic and changing process of cognitive interaction comprising a comprehensive and complex collection of intertwined factors, such as member attributes and interactions. This study only focused on the relationship between the disciplinary diversity of team members and the outcome of collaboration which reflected in societal impact. It remains to be seen how disciplinary diversity affects the innovation performance of collaboration and how organisations can respond to the “marginal dilemma” identified in the present study. Future research should explore how to effectively control the decline in costs and creativity, while maximising team members’ innovative behaviour.

## Methods

### Data

*Nature* and *Science* are multidisciplinary journal, and choosing papers from the same journal in the same year would exclude the effect of different impact factors and years of publication. Thus, we conducted a search of the Web of Science Core Collection which was limited to the journal *Nature and Science*, the publication date “2018” and the literature type “Article”, giving a total of 1596 articles. The data collection process was as followed. First, the basic information of each article was obtained, such as the DOI, authors, institutions, publication date, and so on. Second, using the DOI of each article, the number of mentions on Twitter and the overall altmetrics score was obtained from altmetric.com. The above data were crawled with the help of the altmetrics package for Python (version 3.6.6) for 3 August 2022.

To avoid missing data and ensure the matching of information in the literature, the following papers were excluded: retracted papers; papers with missing information, such as authors’ institutions; papers that was missing altmetrics data; single-author papers; or papers with unclear information on the discipline covered by the primary or secondary institution. The final valid sample comprised 1554 papers.

### Variables

The variables used in this study were defined in Table 4. The dependent variable was societal impact. The independent variable was diversity in interdisciplinary collaboration. The control variables were the date of publication, institutions, and number of authors. The specifics of societal impact, interdisciplinary collaboration diversity, and control variables were detailed below.

**Table 4:** Measures of variables.

Variables	Codes	Measures
**Dependent variable**		
Societal impact	Tweets mentioned count	The number of mentions by Tweets
**Independent variable**		
Interdisciplinary collaboration diversity	Subject count	The number of disciplines involved per publication
**Control variable**		
Institution	Institution count	The number of research institutions involved per publication
Author	Author count	The number of authors involved per publication
Journal	Journal	If the journal is *Nature*, marked as 0; if *Science*, marked as 1
Time lag	Time lag	Time lag between altmetric data acquisition and paper publication (measured in days)
Field	Field	If the publication relates to a main field, the corresponding variable is marked as 1 and vice versa

#### Societal impact

The dependent variable was the societal impact of each paper. To measure it, we adopted the main altmetrics indicator, namely the number of mentions on Twitter^62^. Twitter, as a major public social media platform, broadens the audience and dissemination channels of academic research findings and helps to improve the understanding and application of academic research by the public^63^.

#### Interdisciplinary collaboration diversity

**Figure 3:**
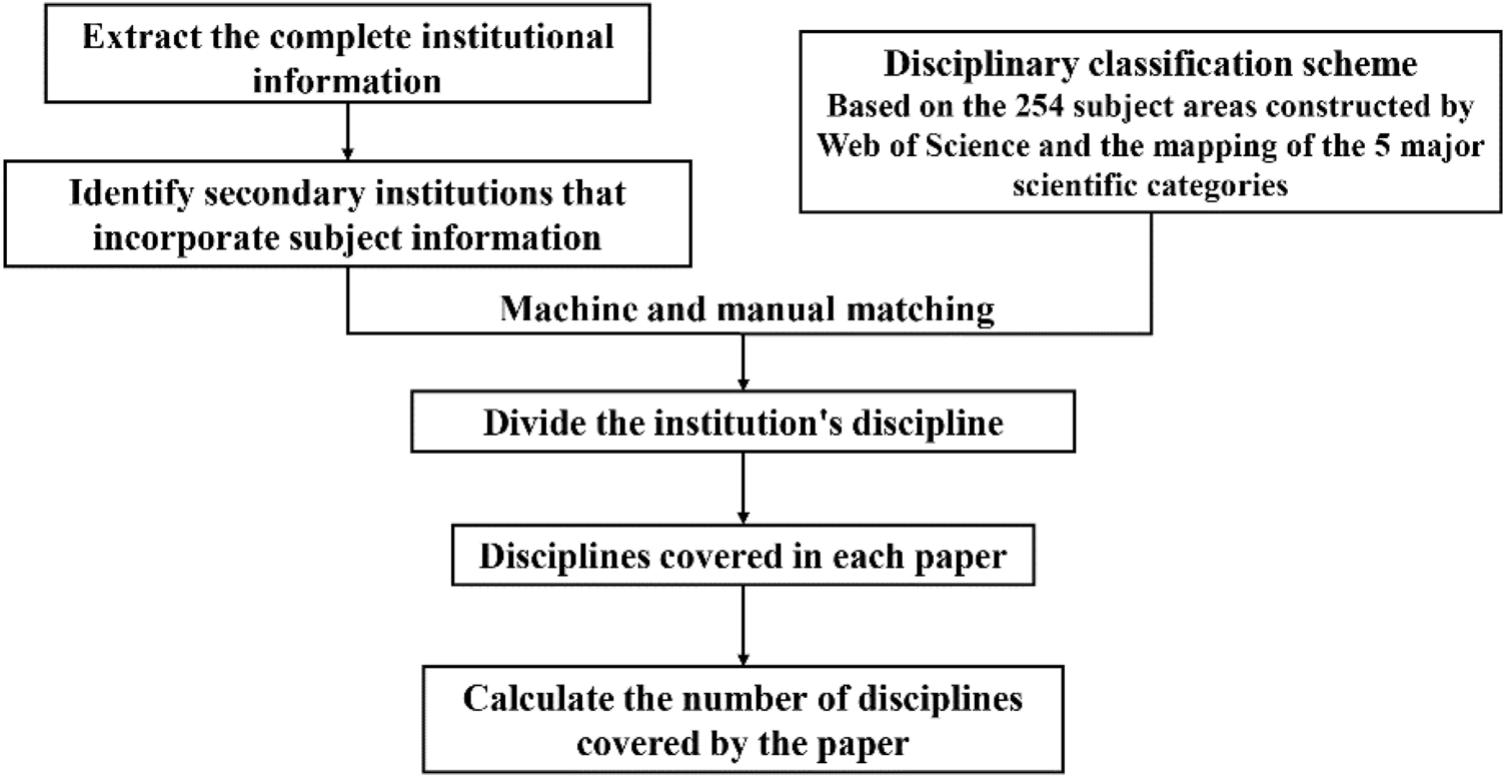
Procedure for measuring knowledge diversity in interdisciplinary research.

Referring to the studies of Zhang et al.^31^ as well as Zhang and Zhang^64^, we obtained the number of disciplines involved in each study to measure the diversity of interdisciplinary collaboration from a scientific activity perspective, starting with information on each author’s institution. Data processing was conducted as followed (Fig. 3). First, the complete address of each institution was extracted, and the characteristic words of the secondary institution were identified, which included information on the discipline. If the secondary institution did not show subject information, the characteristic words of the primary institution were identified. Second, a disciplinary classification scheme was constructed based on the 254 disciplinary categories of the Web of Science and their corresponding five scientific disciplines (Life Sciences & Biomedicine, Technology, Arts & Humanities, Social Sciences, and Physical Sciences). In addition, the subject information words of each institution were matched to the subject categories in the subject classification scheme. Non-English words or ambiguous abbreviations and acronyms were manually checked to ensure accuracy. Following this method, we were able to count the number of different disciplines involved in each publication.

#### Control variables

This study controlled for the number of authors, institutions and the type of journal; it was suggested that these variables have a potential correlation with the academic impact of scientific papers^30^. Considering the real-time nature of altmetric data facilitated by online research communication, we obtained the difference between the date of publication and obtaining altmetric data. This helped control for the effect of the differences in the times of publication of the papers in the sample. Due to the wide variation in the societal attention to research on different topics^65^, this study used subject classification data from the Leiden ranking, i.e., flagging one or more of the main fields covered by the publication. The fields involved were respectively: Biomedical and health sciences, Life and earth sciences, Mathematics and computer science, Physical sciences and engineering and Social sciences and humanities. We included in our analysis the coverage of each of these fields.

## Data Availability

The datasets used or analysed during the current study available from the corresponding author on reasonable request.
