# Author Correction: The inverted U-shaped relationship between knowledge diversity of researchers and societal impact

**DOI:** 10.1038/s41598-022-25513-7

**Published:** 2022-12-02

**Authors:** Gaofeng Wang, Yetong Gan, Haodong Yang

**Affiliations:** School of Humanities and Social Sciences, University of Science and Technology of China, Hefei, China

Correction to: *Scientific Reports* 10.1038/s41598-022-21821-0, published online 03 November 2022

The original version of this Article contained errors in Tables 2 and 3.

In Table 2, values for Wald chi^2^, AIC/BIC, Pseudo R^2^, Alpha 95%CI in the “Independent variables” section were incorrectly given in columns Model 1: *M,* Model 1: *SE,* and Model 2: *M,* instead of in columns Model 1, Model 2 and Model 3. The correct and incorrect values appear below.

Incorrect:

**Table:** 

Variables	Model 1		Model 2		Model 3	
	*M*	*SE*	*M*	*SE*	*M*	*SE*
**Independent variables**						
Wald chi^2^	267.85***	283.70***	295.44***			
AIC/BIC	18420.86/18479.58	18418.83/18482.89	18410.12/18479.52			
Pseudo R^2^	0.024	0.024	0.025			
Alpha 95%CI	[0.941, 1.086]	[0.939, 1.084]	[0.934, 1.078]			

Correct:

**Table:** 

Variables	Model 1		Model 2		Model 3	
	*M*	*SE*	*M*	*SE*	*M*	*SE*
**Independent variables**						
Wald chi^2^	267.85***		283.70***		295.44***	
AIC/BIC	18420.86/18479.58		18418.83/18482.89		18410.12/18479.52	
Pseudo R^2^	0.024		0.024		0.025	
Alpha 95%CI	[0.941, 1.086]		[0.939, 1.084]		[0.934, 1.078]	

In Table 3, values for AIC/BIC, R^2^, and Root MSE in the “Independent variables” section were incorrectly given in columns Model 1: *β*, Model 1: *SE*, and Model 2: *β,* instead of in columns Model 1, Model 2 and Model 3. The correct and incorrect values appear below.

Incorrect:

**Table:** 

Variables	Model 1		Model 2		Model 3	
	*ß*	*SE*	*ß*	*SE*	*ß*	*SE*
**Independent variables**						
AIC/BIC	5782.29/5835.68	5782.253/5840.98	5769.82/5833.89			
R^2^	0.202	0.203	0.211			
Root MSE	1.578	1.578	1.571			

Correct:

**Table:** 

Variables	Model 1		Model 2		Model 3	
	*ß*	*SE*	*ß*	*SE*	*ß*	*SE*
**Independent variables**						
AIC/BIC	5782.29/5835.68		5782.253/5840.98		5769.82/5833.89	
R^2^	0.202		0.203		0.211	
Root MSE	1.578		1.578		1.571	

The original Article has been corrected.

